# A comparison of physiological and perceptual responses to fixed‐power and perceptually regulated cycling with and without blood flow restriction in trained cyclists

**DOI:** 10.1002/ejsc.12068

**Published:** 2024-03-06

**Authors:** Nathan D. W. Smith, Olivier Girard, Brendan R. Scott, Jeremiah J. Peiffer

**Affiliations:** ^1^ Centre for Healthy Ageing Murdoch University Perth Western Australia Australia; ^2^ Murdoch Applied Sports Science Laboratory Discipline of Exercise Science Murdoch University Perth Western Australia Australia; ^3^ School of Human Sciences (Exercise and Sport Science) The University of Western Australia Perth Western Australia Australia

**Keywords:** discomfort, effort, moderate‐intensity, pain, self‐paced, vascular occlusion

## Abstract

This study compared physiological and perceptual responses between cycling prescribed using fixed‐power (PWR) and fixed rating of perceived exertion (RPE), when performed with blood flow restricted (BFR_PWR_ and BFR_RPE_) and unrestricted (CON_PWR_ and CON_RPE_). Endurance cyclists/triathletes cycled for 10 min in four separate randomized conditions; that is, two methods of prescribed exercise intensity (power at the first ventilatory threshold or RPE matched to CON_PWR_) combined with two occlusion levels (with BFR or without). Cardiorespiratory and perceptual variables were recorded every 2 min. Blood lactate concentration was measured pre‐, immediately and 2‐min postexercise. Power output during BFR_RPE_ was lower than CON_RPE_ (−13 ± 13%). The greatest physiological and perceptual responses were achieved during BFR_PWR_. Heart rate during BFR_RPE_ was not different compared with CON_PWR_, yet was greater than CON_RPE_ (+4 ± 11%). Muscular discomfort during BFR_RPE_ was greater than CON_PWR_ (+43 ± 18%) and CON_RPE_ (+65 ± 58%). Cuff pain was greater during BFR_PWR_ than BFR_RPE_ (+14 ± 21%). Blood lactate concentration was not different between BFR_RPE_, CON_PWR_, and CON_RPE_ at any timepoint. The reduction in power (fixed‐RPE trials; BFR *minus* unrestricted) correlated with changes in the respiratory rate (*r* = 0.85, confidence intervals [CI] = 0.51, 0.96) and postexercise lactate (*r* = 0.75, CI = 0.27, 0.93) but not muscular discomfort (*r* = 0.18, CI = −0.47, 0.71). Cardiorespiratory and metabolic stress, muscular discomfort, and cuff pain likely mediated self‐regulating fixed‐RPE cycling with BFR. While cycling with BFR at a fixed‐RPE resulted in less physiological stress compared to BFR_PWR_, it still provided a heightened level of physiological stress, with less pain and discomfort. As such, fixed‐RPE can be a suitable alternative for prescribing BFR to trained cyclists.

## Highlights

- Applying BFR at the fixed‐power associated with the first ventilatory threshold can increase the heart rate from the moderate to high exercise intensity domain for some individuals, indicating BFR exercise may not necessarily be low intensity.
- Fixed‐power cycling with BFR can be used at low work rates, perhaps following injury, yet practitioners should consider the balance between the physiological demands, discomfort, and pain at any given work rate.
- Discomfort and pain associated with BFR is alleviated in a fixed‐RPE model, which presents a suitable method of prescribing aerobic BFR cycling, yet physiological stress is lower compared to BFR cycling at a fixed‐power.

## 1 INTRODUCTION

Applying blood flow restriction (BFR) during low‐intensity exercise training (i.e., below the first lactate threshold) has been shown to improve some physiological determinants of endurance performance in trained endurance athletes. For example, 5 weeks of rowing with BFR applied to the thighs has increased the maximal oxygen uptake of trained rowers by 9.1% (baseline = 63.0 mL·kg^−1^ min^−1^) with no improvement shown in the group training without BFR (both groups rowed at an intensity consistent with <2 mmol·L^−1^ blood lactate measured in a non‐BFR state) (Held et al., 2020). This increase in highly trained endurance athletes (Held et al., 2020) was likely due to the unique stimulus BFR presents (Ferguson et al., 2021; Smith et al., 2021). For example, low‐intensity training with BFR can increase femoral artery diameter (Christiansen et al., 2020), capillary density (Nielsen et al., 2020), and buffering capacity (Christiansen et al., 2021); findings that are not observed during training at the same absolute work rate without BFR. Endurance athletes could therefore use BFR to stimulate similar physiological adaptations compared to high‐intensity interval training without the high mechanical load (Ferguson et al., 2021). This could be useful for healthy athletes in periods of high volume training, to manage training load, or for injured athletes in rehabilitation, as an initial stimulus to improve functional outcomes and perhaps shorten the time until they return to peak performance (Cognetti et al., 2022). Low‐to‐moderate intensity cycling with BFR could therefore be an effective and complimentary training stimulus for endurance cyclists with training needs.

Aerobic BFR cycling is commonly prescribed using intensities fixed at a percentage of the individual's maximal capacity (e.g., 40% peak power output) (Thomas et al., 2018). However, this approach is likely problematic as relative physiological thresholds are not directly comparable between exercises with and without BFR (Ozaki et al., 2010; Sakamaki‐Sunaga et al., 2012). Indeed, heart rate is a commonly used method of prescribing training intensity (Ferguson, 2014), yet application of BFR substantially increases the heart rate (+20 bpm) while exercising at relatively low intensities (Ozaki et al., 2010). The use of self‐paced exercise prescription may alleviate this issue as it does not rely on metrics such as the heart rate or power output (Seiler & Sjursen, 2004). Nevertheless, self‐paced exercise is reliant on exercise‐related sensations (Abbiss et al., 2015) such as rating of perceived exertion (RPE), effort, and pain to self‐regulate intensity, which can be influenced by BFR. For example, BFR elevates RPE during fixed‐power cycling compared to unrestricted exercise at the same external work rate (Thomas et al., 2018). The increased RPE is likely due to heightened physiological demands as well as muscular discomfort and pain from the BFR cuffs (Borg et al., 1985). Indeed, greater leg pain has been reported cycling at 40% maximal oxygen uptake with BFR (∼2.5 out of 10 au) compared to without BFR (no pain reported), which corresponded with a concomitantly greater RPE (∼8.9 au and ∼6.1 au [6–20 scale], respectively) (Kilgas et al., 2022). Considering the numerous factors impacting on perceptual sensations during exercise, BFR could interfere with an athlete's ability to self‐regulate exercise intensity. The inability to maintain a constant physiological intensity (i.e., self‐regulate) during exercise with BFR could result in lower overall demands compared to a constant work rate model. Thus, these prescription methods should be compared to determine if a self‐regulated approach is suitable for prescribing aerobic BFR cycling to trained cyclists.

The purpose of this study was to compare the performance (power output), perceptual (RPE, effort, discomfort, and pain), and physiological (heart rate, respiratory rate, and oxygen uptake (V̇O_2_)) responses to cycling with and without BFR using both fixed‐work rate and fixed‐RPE exercise prescription models. It was hypothesized that applying BFR during fixed‐RPE trials would elevate perceptual responses (excluding RPE) and lower power output without altering physiological demands compared to fixed‐RPE without BFR. Additionally, fixed‐power cycling without BFR would produce lower perceptual responses (excluding RPE) with no difference in physiological demands compared to cycling at the same RPE with BFR.

## 2 METHODS

Twelve trained male endurance cyclists/triathletes were recruited (age: 40 ± 11 year; body mass: 75.7 ± 5.8 kg; stature: 178.0 ± 4.9 cm; maximal oxygen uptake: 52.8 ± 3.6 mL·kg^−1^ min^−1^; peak erobic power: 372 ± 43 W; and training volume: 193 ± 78 km·week^−1^). Training status was defined using a maximal cycling test to exhaustion and established criteria (De Pauw et al., 2013). The study's purpose and requirements were explained to participants before obtaining written informed consent. Individuals were excluded if they indicated hematological, musculoskeletal, or neuromuscular abnormalities, or were taking medications likely to influence the main outcome measures. Ethical approval was obtained from the institutional ethics committee (ref:2021/054). Females were not included in this study due to their reported higher sensitivity to experimentally induced (including ischemic) pain compared to men (Bartley & Fillingim, 2013).

Participants first completed a preliminary visit (determination of BFR pressure, incremental cycling test to exhaustion, and familiarization with BFR) before four experimental sessions, which were all separated by at least 24 h and conducted at the same time of day (±2 h). Experimental sessions involved cycling for 10 min using two methods of prescribing exercise intensity, fixed‐power (PWR) and fixed‐RPE, each with and without BFR (BFR and CON): BFR_PWR_, CON_PWR_, BFR_RPE_, and CON_RPE_. The RPE prescribed during BFR_RPE_ and CON_RPE_ was matched to the RPE reported during CON_PWR_. The experimental design therefore required CON_PWR_ to be performed first, with the three remaining experimental conditions completed in a randomized, counterbalanced order. Participants were asked to refrain from alcohol, caffeine, and strenuous exercise during the 24 h prior to each visit.

Upon arrival to the preliminary session, participants rested supine for 10 min to determine the individualized BFR pressure. Participants then performed a 5‐min self‐selected warm‐up at a freely adjustable work rate followed by an incremental cycling test. The test involved 1‐min stages beginning at 70 W with a work rate increase of 35 W·min^−1^, and ended at volitional exhaustion or when the self‐selected cadence dropped below 60 rpm for 5 s. Ventilatory gases were measured using a metabolic cart (TrueOne 2400, ParvoMedics). Maximal oxygen uptake was calculated as the average of the two highest consecutive 15‐s mean values. Two exercise physiologists independently determined the first and second ventilatory thresholds; (VT_1_ and VT_2_): VT_1_ was identified as a sudden rise in V̇E/V̇O_2_ with no increase in V̇E/V̇CO_2_, while VT_2_ was determined using the criteria of an exponential increase in both V̇E/V̇O_2_ and V̇E/V̇CO_2_ (Lucía et al., 2000). Discrepancies between physiologists was resolved by consulting a third assessor. Peak power output was calculated as the power of the last completed stage plus a pro rata value of the final stage (Peiffer et al., 2008). Finally, participants were familiarized to experimental procedures using 10 min of self‐paced cycling with BFR. Cycling was performed using a Velotron ergometer (RacerMate, USA).

During experimental sessions, participants warmed‐up for 5‐min at 50% of the power output associated with VT_1_ (108 ± 13 W), rested passively for 3 min, and then completed the 10‐min cycling bout. Power output during the warm‐up of each experimental session and the 10‐min bout for both BFR_PWR_ and CON_PWR_ (at the power associated with VT_1_; 217 ± 27 W) were maintained using Velotron software. Participants reported RPE, effort, muscular discomfort, and cuff pain every 2 min during the 10‐min bout (explained in the following section). During BFR_RPE_ and CON_RPE_, participants were instructed to cycle at the RPE they reported at the end of the same 2‐min period during CON_PWR_, which was also visually represented on a CR‐10 scale positioned in front of the bike (0–2 min = 3.1 ± 0.5 au, 2–4 min = 3.3 ± 0.5 au, 4–6 min = 3.4 ± 0.5 au, 6–8 min = 3.7 ± 0.7 au, and 8–10 min = 3.6 ± 0.7 au). To allow participants to cycle at a given RPE, the work rate was freely adjustable. Participants were asked to maintain the same constant self‐selected cadence produced during CON_PWR_, and thus were blind to all measurements except the cadence and time elapsed. Fingertip blood lactate concentration was measured 15 s prior, immediately after, and 2 min following the 10‐min bout using a handheld analyzer (Lactate Pro II, Arkray, Japan). Power output was measured using a power meter (InfoCrank, Verve Cycling, Australia) fitted to the Velotron. The heart rate (HRM‐Dual, Garmin, USA) and power output were recorded using a cycling computer (130 Edge, Garmin, USA). Both V̇O_2_ and respiratory rate were measured using the metabolic cart. Data were averaged into 2‐min mean values. A fan 1 m in diameter producing a wind speed of 32 km·h^−1^ was placed 2 m in front of the bike.

Participants' RPE, perceived effort, muscular discomfort, and cuff pain were obtained using separate 11‐point numeric scales ranging from 0 “Nothing at all” to 10 “Maximal”, except effort, which ranged from 0% “Nothing at all” to 100% “Maximal”. Borg's CR‐10 (Borg, 1982) scale was used to obtain RPE, with all others constructed using the same numbers and similar anchors (i.e., 3 = “*Moderate*”) as the CR10. Each perceptual scale was explained during familiarization and the definition of each metric restated at the beginning of each experimental session. Participants were instructed that RPE was “*a measure of whole‐body physical exertion and should encompass cardiovascular demands, or the sense of “breathlessness”, as well as sensations in the muscles of the legs caused by exercise and other sensations associated with exertion*” (Peñailillo et al., 2018). Perceived effort was defined as “*the amount of mental or physical energy being given to complete the task. It is the overall effort needed to maintain the intensity of the exercise*” (du Plessis et al., 2020). Muscular discomfort was defined as “*any uncomfortable sensation within the leg muscles associated with exercise.*”. Cuff pain was defined as “*the intensity of pain experienced specifically from the BFR cuffs compressing your thigh. This includes any type of pain, such as sharp, dull, or throbbing pain.*”

Individualized BFR pressures were based on estimates of arterial occlusion pressure using an established equation (Loenneke et al., 2015) incorporating resting measurements of thigh circumference and blood pressure (HEM‐7203, Omron, Australia). During BFR sessions, 5‐cm wide pneumatic cuffs (5CS, Hokanson, USA) were applied to the proximal aspect of the thighs. The cuffs were instantaneously inflated (E20 inflator and AG101 air source, Hokanson, USA) to 60% of arterial occlusion pressure (173 ± 12 mmHg) immediately before the 10‐min bout. Arterial occlusion was estimated as the Hokanson system could not produce sufficient pressure to induce arterial occlusion with 5‐cm cuffs during piloting, and wider cuffs were reported by participants to impact on their cycling technique (Smith, Peiffer, Girard, & Scott, 2022).

During data collection, one participant was unable to complete the BFR_PWR_ session resulting in the removal of their entire data set from all further analyses. Linear mixed models were used to examine differences for all variables, with the participant included as a random factor. Models used to examine differences in power output during fixed‐RPE trials and RPE during fixed‐power trials included fixed effects of BFR (two levels: with and without) and time (five levels: 2, 4, 6, 8, and 10 min). The model for pain during BFR trials included the fixed effects of time (five levels: 2, 4, 6, 8, and 10 min) and prescription method (two levels: fixed‐power and fixed‐RPE). Three‐factor models to examine differences in V̇O_2_, respiratory rate, heart rate, blood lactate, effort, and muscular discomfort included BFR (levels: with and without), time (five levels: 2, 4, 6, 8, and 10 min), and prescription method (two levels: fixed‐power and fixed‐RPE). Main and interaction effects were examined using the Holm–Bonferroni method. Effect sizes were calculated as Cohen’s *d*_z_ using mean values of the session, timepoint, or condition as appropriate. Pearson correlation coefficients with 95% confidence intervals (CI: lower limit and upper limit) were used to examine the association between the difference (BFR *minus* unrestricted) of all variables and RPE during fixed‐power trials or power output during fixed‐RPE trials. Analyses were performed using jamovi (v2.0.0) with significance ≤0.05. Data are presented as mean ± standard deviation. Values are presented as mean percent change ± standard deviation of the percent change (BFR *minus* unrestricted).

## 3 RESULTS

Power output (Table 1; two‐way analysis between BFR_RPE_ and CON_RPE_) was 13 ± 13% lower during BFR_RPE_ (177 ± 40 W) compared to CON_RPE_ (203 ± 35 W; *d*_z_ = 1.0) and increased over time without interaction.

**TABLE 1:** Power output and perceptual responses during 10 min of cycling at a fixed‐power (PWR) and fixed‐rating of perceived exertion (RPE) both with blood flow restriction (BFR) and without (CON).

		Timepoint					*p*‐value						
Variable	Condition	2 min	4 min	6 min	8 min	10 min	BFR	Time	P.Method	BFR × Time	BFR × P.Method	Time × P.Method	BFR × Time × P.Method
Power output (W)	BFR_RPE_ ^g^	162 ± 48	179 ± 40^a^	186 ± 40^a^	181 ± 33^a^	176 ± 39^a^	**<0.001**	**<0.001**	‐	0.395	‐	‐	‐
	CON_RPE_	178 ± 37	198 ± 42	213 ± 34	217 ± 22	211 ± 29							
RPE (0–10 au)	BFR_PWR_	3.4 ± 0.7	4.0 ± 0.4	4.7 ± 0.9^a^ ^,^ ^b^	5.4 ± 1.2^a^ ^,^ ^b^	5.8 ± 1.4^a^ ^,^ ^b^ ^,^ ^c^	**<0.001**	**<0.001**	‐	**<0.001**	‐	‐	‐
	CON_PWR_	3.1 ± 0.5	3.2 ± 0.4	3.5 ± 0.5^d^	3.7 ± 0.6^d^	3.6 ± 0.7^d^							
Effort (%)	BFR_PWR_	33 ± 8	38 ± 9	46 ± 11^a^	55 ± 16^a^ ^,^ ^b^	56 ± 16^a^ ^,^ ^b^ ^,^ ^c^	**<0.001**	**<0.001**	**<0.001**	**0.004**	**<0.001**	0.061	**0.028**
	CON_PWR_	28 ± 6	30 ± 5	31 ± 5^d^	35 ± 7^d^	36 ± 7^d^							
	BFR_RPE_	30 ± 6	32 ± 4	37 ± 5	38 ± 6^d^	40 ± 9^d^							
	CON_RPE_	26 ± 5	29 ± 5	33 ± 5^d^	35 ± 5^d^	35 ± 8^d^							
Muscular discomfort (0–10au)	BFR_PWR_ ^e^ ^,^ ^f^ ^,^ ^g^	3.0 ± 0.8^h^	3.7 ± 0.6^h^	4.4 ± 1.1^h^ ^,^ ^i^	5.0 ± 1.3^h^ ^,^ ^i^	5.6 ± 1.6^h^	**<0.001**	**<0.001**	**<0.001**	**<0.001**	**0.002**	0.885	0.124
	CON_PWR_	2.1 ± 0.7	2.4 ± 0.5	2.8 ± 0.8	2.6 ± 0.7	2.8 ± 0.9							
	BFR_RPE_ ^e^ ^,^ ^g^	2.6 ± 0.9	3.0 ± 0.6	3.6 ± 0.7	4.4 ± 0.8	4.4 ± 0.8							
	CON_RPE_	1.5 ± 0.7	2.3 ± 0.8	2.6 ± 0.9	2.6 ± 0.8	2.9 ± 0.8							
Cuff pain (0–10 au)	BFR_PWR_ ^f^	3.2 ± 0.6	4.2 ± 0.8^a^	4.8 ± 1.3^a^ ^,^ ^b^	5.6 ± 1.9^a^ ^,^ ^b^ ^,^ ^c^	6.0 ± 1.9^a^ ^,^ ^b^ ^,^ ^c^	‐	**<0.001**	**<0.001**	‐	‐	0.635	‐
	BFR_RPE_	2.6 ± 1.0	3.5 ± 0.8	4.2 ± 0.8	4.6 ± 0.9	4.7 ± 0.8							

*Note*: Fixed‐power was set at the power associated with the first ventilatory threshold (217 ± 27 W). Fixed‐RPE trials were prescribed at the same RPE reported at each 2‐min period during CON_PWR_. Data presented as mean ± standard deviation. Significant *p*‐values (<0.05) are highlighted in bold.

Abbreviations: BFR_PWR_, fixed‐power with BFR; BFR_RPE_, fixed‐RPE with BFR; CON_PWR_, fixed‐power without BFR; CON_RPE_, fixed‐RPE without BFR; P.Method, prescription method.

^a^ Different to 2 min.

^b^ Different to 4 min.

^c^ Different to 6 min.

^d^ Different to BFR_PWR_.

^e^ Different to CON_PWR_.

^f^ Different to BFR_RPE_.

^g^ Different to CON_RPE_.

^h^ BFR different to without BFR.

^i^ Effect of BFR, different to previous timepoint.

**FIGURE 1:**
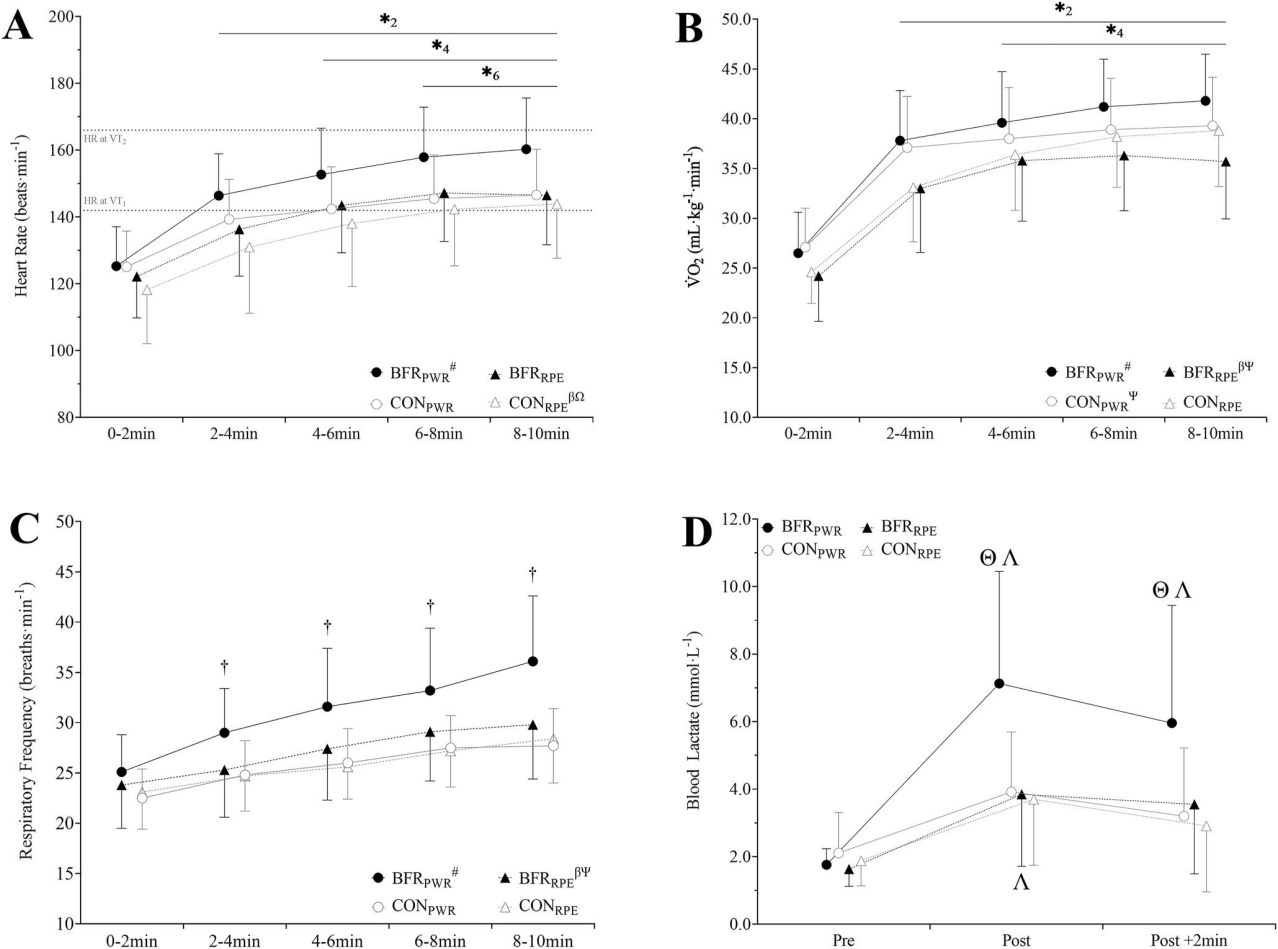
Mean 2‐min heart rate (A), oxygen consumption (V̇O_2_; B), and respiratory frequency (C) during 10 min of cycling, as well as blood lactate concentration 15 s prior (pre), immediately after (post), and 2 min after (post +2 min) exercise (D). Four conditions were completed, involving 10‐min of cycling: with blood flow restriction (BFR) and without (CON) at a fixed power (BFRPWR and CONPWR) and rating of perceived exertion (BFRRPE and CONRPE) matched to CONPWR. ^#^different to all other conditions; ^β^different to CONPWR; ^Ω^different to BFRRPE; ^Ψ^different to CONRPE; *denotes a main effect of time, different from minute two (2), four (4), or six (6); ^†^denotes a time by occlusion interaction, different between BFR and unrestricted; and ^Θ^BFRPWR different to all other conditions at that timepoint; ^Λ^different to pre.

An interaction was observed for the heart rate (Figure 1A) between BFR application (i.e., with BFR versus unrestricted) and the prescription method (i.e., fixed‐power versus fixed‐RPE; *p* = 0.014); however, a three‐way interaction (BFR by time by the prescription method) was not observed (*p* = 0.077). A main effect of time was also noted, with the heart rate increasing across the trials (*p* < 0.001). The heart rate was greater during BFR_PWR_ (149 ± 18 beats·min^−1^) compared to all other conditions (CON_PWR_:140 ± 14 beats·min^−1^, *p* < 0.001, and *d*_z_ = 1.8; BFR_RPE_:139 ± 16 beats·min^−1^, *p* < 0.001, and *d*_z_ = 1.0; and CON_RPE_:135 ± 19 beats·min^−1^, *p* < 0.001, and *d*_z_ = 1.6). The heart rate was 4 ± 7% higher for CON_PWR_ than CON_RPE_ (*p* < 0.001, *d*_z_ = 0.6), and 4 ± 10% greater for BFR_RPE_ compared to CON_RPE_ (*p* < 0.001 *d*_z_ = 0.4).

An interaction was observed for V̇O_2_ between BFR application and the prescription method (*p* < 0.001; Figure 1B), without a three‐way interaction (*p* = 0.102). A main effect of time was also noted, with V̇O_2_ increasing across trials (*p* < 0.001). Greater V̇O_2_ was observed during BFR_PWR_ (37.4 ± 7.3 mL·kg^−1^ min^−1^) compared to all other conditions (CON_PWR_:36.1 ± 6.5 mL·kg^−1^ min^−1^, *p* = 0.029, and *d*_z_ = 0.6; CON_RPE_:34.2 ± 7.1 mL·kg^−1^ min^−1^, *p* < 0.001, and *d*_z_ = 0.8; BFR_RPE_:33.0 ± 7.2 mL·kg^−1^ min^−1^, *p* < 0.001, and *d*_z_ = 1.0). Additionally, CON_PWR_ was greater compared to CON_RPE_ by 6 ± 11% (*p* = 0.002, *d*_z_ = 0.5) and BFR_RPE_ by 10 ± 12% (*p* < 0.001, *d*_z_ = 0.8). CON_RPE_ was 5 ± 12% greater compared to BFR_RPE_ (*p* = 0.029, *d*_z_ = 0.3).

Two‐way interactions were observed for the respiratory rate with differences noted between BFR application and the prescription method (*p* < 0.001; Figure 1C) as well as between BFR application and time (*p* = 0.021), without a three‐way interaction (*p* = 0.205). The respiratory rate was greater during BFR_PWR_ (31±6breaths·min^−1^) compared to all other conditions (CON_PWR_:26 ± 4 breaths·min^−1^, *p* < 0.001, and *d*_z_ = 1.4; CON_RPE_:26 ± 4 breaths·min^−1^, *p* < 0.001, and *d*_z_ = 1.7; BFR_RPE_:27 ± 5 breaths·min^−1^, *p* < 0.001, and *d*_z_ = 0.9), and BFR_RPE_ was 5 ± 10% greater compared to both CON_PWR_ (*p* = 0.010, *d*_z_ = 0.6) and CON_RPE_ (*p* = 0.013, *d*_z_ = 0.5). Additionally, the respiratory rate was greater with BFR compared to without BFR at minutes two to four (27 ± 5 breaths·min^−1^ versus 25 ± 3 breaths·min^−1^, *p* = 0.027, and *d*_z_ = 1.7), four to six (29 ± 6 breaths·min^−1^ versus 26 ± 3 breaths·min^−1^, *p* < 0.001, and *d*_z_ = 1.3), six to eight (31 ± 6 breaths·min^−1^ versus 27 ± 3 breaths·min^−1^, *p* < 0.001, and *d*_z_ = 1.4), and eight to ten (33 ± 7 breaths·min^−1^ versus 28 ± 4 breaths·min^−1^, *p* < 0.001, and *d*_z_ = 1.9).

A three‐way interaction was observed for blood lactate concentration (*p* = 0.039; Figure 1D). Lactate concentrations during BFR_PWR_ were greater compared to all other conditions immediately postexercise (CON_PWR_:90 ± 67%, *p* < 0.001, and *d*_z_ = 1.4; BFR_RPE_:115 ± 93%, *p* < 0.001, and *d*_z_ = 1.2; CON_RPE_:129 ± 149%, *p* < 0.001, and *d*_z_ = 1.2) and 2 min postexercise (CON_PWR_:90 ± 53%, *p* = 0.003, and *d*_z_ = 1.6; BFR_RPE_:97 ± 97%, *p* = 0.029, and *d*_z_ = 0.8; and CON_RPE_:137 ± 120%, *p* < 0.001, and *d*_z_ = 1.4). No differences were observed for pre‐exercise lactate measures.

Perceptual responses and the associated *p*‐values for main effects are shown in Table 1. An interaction was observed for RPE, with an increase over time only during BFR_PWR_, and greater values reported during BFR_PWR_ compared to CON_PWR_ at minute six (*p* = 0.001, *d*_z_ = 1.4), eight (*p* < 0.001, *d*_z_ = 1.5), and ten (*p* < 0.001, *d*_z_ = 1.6). Perceived effort increased over time only during BFR_PWR_, and was greater at minutes eight and 10, compared to all other conditions (CON_PWR_:*p* < 0.001, *d*_z_ = 1.3 and *p* < 0.001, *d*_z_ = 1.7; BFR_RPE_:*p* < 0.001, *d*_z_ = 1.4 and *p* < 0.001, *d*_z_ = 1.4; and CON_RPE_:*p* < 0.001, *d*_z_ = 1.2 *p* < 0.001, *d*_z_ = 1.5). Muscular discomfort increased over time for BFR trials only, and was greater compared to unrestricted at all timepoints (minute two: 2.8 ± 0.9 au versus 1.8 ± 0.8 au, *p* < 0.001, and *d*_z_ = 1.6; four: 3.4 ± 0.7 au versus 2.3 ± 0.7 au, *p* < 0.001, and *d*_z_ = 2.2; six: 4.0 ± 1.0 au versus 2.7 ± 0.8 au, *p* < 0.001, and *d*_z_ = 1.7; eight: 4.7 ± 1.3 au versus 2.7 ± 0.7 au, *p* < 0.001, *d*_z_ = 3.1; and ten: 5.0 ± 1.4 au versus 2.9 ± 0.8 au, *p* < 0.001, and *d*_z_ = 2.9). Cuff pain was greater during BFR_PWR_ compared to BFR_RPE_ (2.4 ± 2.7 au versus 2.0 ± 2.1 au, *p* = 0.016, and *d*_z_ = 0.8) and increased throughout both trials.

**FIGURE 2:**
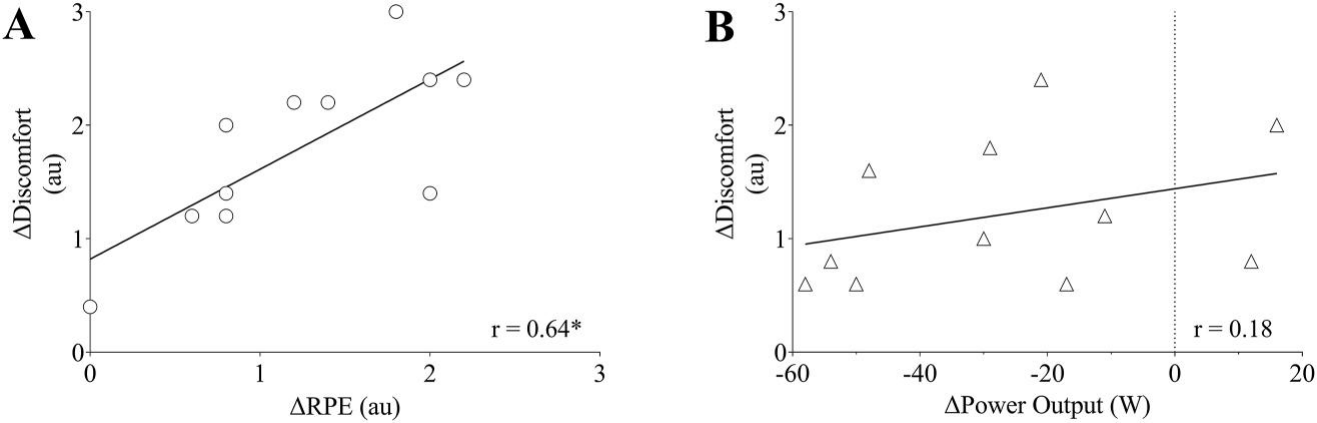
Correlations between the difference in average muscular discomfort versus (A) difference in average rating of perceived exertion (RPE) during fixed‐power trials, and (B) difference in average power output during fixed‐RPE trials. Difference scores are calculated by subtracting the trial with blood flow restriction from the trial without restriction. *significant correlation (*p* = 0.036).

For fixed‐power trials, the change in RPE (BFR *minus* unrestricted) was correlated with blood lactate 2 min postexercise (*r* = 0.65; CI = 0.08,0.90; and *p* = 0.031) and muscular discomfort (Figure 2A, *r* = 0.64; CI = 0.06,0.90; and *p* = 0.036). The RPE was not correlated with V̇O_2_ (*r* = −0.50; CI = −0.85, 0.14; and *p* = 0.117), respiratory rate (*r* = 0.51; CI = −0.14, 0.85; *p* = 0.113), heart rate (*r* = 0.60; CI = −0.01, 0.88; and *p* = 0.053), or blood lactate immediately postexercise (*r* = 0.48; CI = −0.17, 0.84; and *p* = 0.133).

For fixed‐RPE trials, the change in power (BFR *minus* unrestricted) was correlated with V̇O_2_ (*r* = 0.88; CI = 0.59, 0.97; and *p* < 0.001), respiratory rate (*r* = 0.85; CI = 0.51, 0.96; and *p* < 0.001), and blood lactate both immediately (*r* = 0.75; CI = 0.27, 0.93; and *p* = 0.008) and 2 min postexercise (*r* = 0.74; CI = 0.25, 0.93; and *p* = 0.008). Power output was not correlated with the heart rate (*r* = 0.21; CI = −0.44, 0.72; and *p* = 0.527) and muscular discomfort (Figure 2B; *r* = 0.18; CI = −0.47, 0.71; and *p* = 0.59).

## 4 DISCUSSION

This study examined performance, physiological, and perceptual responses to 10 min of cycling at a fixed‐power and fixed‐RPE with and without BFR. Comparisons between fixed‐RPE trials showed, in agreement with the first hypothesis, cycling with BFR reduced power output and caused greater muscular discomfort compared to cycling without BFR. Additionally during fixed‐RPE trials, the use of BFR was associated with increased respiratory rate, heart rate, pre‐to‐post exercise blood lactate, and reduced V̇O_2_ without altering perceived effort compared to cycling without BFR. Comparisons between fixed‐RPE with BFR and fixed‐power without BFR showed that in agreement with the second hypothesis, fixed‐RPE cycling with BFR increased muscular discomfort and did not alter the heart rate and the respiratory rate, yet V̇O_2_ was lower compared to fixed‐power without BFR. Cuff pain was greater using fixed‐power compared to fixed‐RPE. These findings indicate BFR alters physiological and perceptual responses to fixed‐power and fixed‐RPE modalities differently, which may be important for prescribing aerobic BFR exercise.

A key aim of the present study was to examine the self‐regulation of the physiological intensity of self‐paced exercise with BFR. Heart rate, respiratory rate, and blood lactate were not different between BFR_RPE_ and CON_RPE_, yet V̇O_2_ was lower during BFR_RPE_ and CON_RPE_. However, due to impaired venous return, indirect calorimetry likely underestimates ventilatory V̇O_2_ measurements (Walden et al., 2022). As such, notwithstanding V̇O_2_ differences, heart rate, respiratory rate, and blood lactate data are indicative that participants successfully autoregulated the physiological stress during BFR_RPE_. To further support this, the time course of changes for cardiorespiratory variables was not different between conditions, with only a main effect of time noted (Figure 1). Furthermore, the physiological responses and power output plateaued in all conditions and did not demonstrate a stochastic response that would indicate participants could not self‐regulate exercise intensity. It is acknowledged that this finding is isolated to a 10‐min bout of exercise. Should this technique be expanded to a longer training session, one may need to consider how prior exercise may impact the BFR exercise bout or how BFR may influence subsequent exercise. Overall, BFR does not interfere with a cyclists' ability to self‐regulate the physiological demands of moderate intensity self‐paced exercise, indicating that a perceptually regulated approach is a suitable method of prescribing aerobic BFR cycling to trained cyclists.

Power output during fixed‐RPE trials (BFR and CON) did not change after the second minute of exercise, yet applying BFR lowered overall power output compared to cycling without restriction (Table 1). Despite the lower power output, both the heart rate and the respiratory rate were greater during fixed‐RPE with BFR compared to without BFR. These findings are likely due to the influence of BFR on venous pooling, thereby leading to an increase in the heart rate (Alam & Smirk, 1938; Ozaki et al., 2010), while BFR‐induced increases in hypoxia and pain have both been shown to increase the respiratory rate (Duranti et al., 1991; Lam et al., 2019). While the noted decline in power output was not unexpected with BFR (Smith, Girard, Scott, & Peiffer, 2022), the decrease is closely associated with pain and muscular discomfort. During the fixed‐RPE trials, the reduced power output likely attenuated the increase in muscular discomfort, as no significant correlation was observed for between‐condition differences in power and muscular discomfort (Figure 2B). A possible explanation could be that participants consciously selected a lower power output, compared to the condition without BFR, to sustain their tolerable magnitude of discomfort. In support of this, during fixed‐power trials the difference in RPE with and without BFR was correlated with the changes in blood lactate 2 min postexercise and muscular discomfort (Figure 2A). Practically, the lower power output associated with BFR could be beneficial within periodization or rehabilitation, yet this outcome is at the sacrifice of elevated discomfort and pain during exercise. The greater localized physiological demands are thereby important in modulating muscular discomfort and subsequent RPE, and these factors contribute to an individual's willingness to maintain a desired power output (Ciubotariu et al., 2004; Cook et al., 1997).

Observing the greatest cardiorespiratory, metabolic, and perceptual demands during fixed‐power cycling with BFR is relevant for prescribing BFR cycling. One third of participants demonstrated heart rates exceeding those corresponding to VT_2_ (measured during the graded exercise test) during fixed‐power with BFR, indicating exercise intensity shifted from the *moderate* (i.e., between VT_1_ and VT_2_) to *heavy* domain (i.e., >VT_2_) (Burnley & Jones, 2007). Importantly, this finding on its own does not indicate participants' metabolic state was typical of exercise in the heavy exercise domain, as BFR increases the heart rate by reducing venous return (Ozaki et al., 2010). It is possible that participants' heart rates were slightly augmented by day‐to‐day variance in the heart rate (Quer et al., 2020), yet other studies have observed larger increases in the heart rate (∼20 beats·min^−1^) when BFR is applied (Ozaki et al., 2010; Thomas et al., 2018). Nevertheless, such increases in relative exercise intensity at the power associated with VT_1_ are still ecologically valid, indicating the heart rate may not be the most appropriate method for prescribing aerobic BFR exercise. The increase in relative physiological intensity was accompanied by an increase in RPE within BFR_PWR_ between minutes two and 10 from *moderate* (∼3.4 au) to *hard/very‐hard* (∼5.8 au) with no change in RPE throughout CON_PWR_ (∼3.4 au). Additionally, within this study one cyclist was able to complete the fixed‐RPE with BFR but not fixed‐power with BFR trial; a finding consistent with other aerobic BFR research (Lauver et al., 2021). This indicates that using fixed intensities to prescribe BFR exercise may not be feasible for all individuals. Therefore, RPE is likely more appropriate compared to power output or the heart rate for prescribing moderate to high intensity exercise with BFR.

Within the literature, prescribing exercise to a set metabolic or ventilatory threshold is a common practice (Stöggl & Sperlich, 2015). While the authors of this paper are not aware of evidence demonstrating day‐to‐day variability in physiological measures when exercising at these thresholds (i.e., VT_1_ used in this study), such variability could exist, as indicated in respect to the heart rate within this discussion. Indeed, under fatigued conditions, such as those experienced by multiple days of training, it is likely that physiological and perceptual response will increase to a given stimuli (Halson et al., 2002). In the present study, prescribing exercise at the power associated with VT_1_ for the CON_PWR_ and BFR_PWR_ conditions may have influenced some of the measured outcomes. It is possible that inherent variability in the physiological responses resulted in some participants exercising in a harder intensity domain, although the alternative should also be considered. While this does present as a limitation to this study, the differences noted within these data, specifically when analyzing conditions against CON_PWR_ or BFR_PWR_, are larger than one would anticipate if solely a consequence of day‐to‐day variability in the physiological and perceptual responses. As such, we believe the study outcomes should be considered as robust within the confines of this limitation.

## 5 CONCLUSION

Cycling at a fixed‐RPE with BFR lowered power output compared to unrestricted, likely to alleviate pain and discomfort, yet cardiorespiratory (heart rate and respiratory rate) and metabolic stress (blood lactate concentrations) was not different between these conditions. Therefore, cyclists can successfully use RPE to self‐regulate the physiological intensity during self‐paced BFR cycling. The reduced power during fixed‐RPE cycling with BFR lowered the cardiovascular demands compared to fixed‐power cycling with BFR. Furthermore, fixed‐power with BFR was associated with the greatest perceptual responses and was not tolerable for one participant. The method of prescribing aerobic BFR exercise is therefore important for the physiological demands and should be considered in relation to the perceptual responses. A fixed‐RPE approach is a convenient method of prescribing moderate intensity aerobic BFR cycling, as athletes can select the same RPE used for sessions of a similar duration without BFR.

## CONFLICT OF INTEREST STATEMENT

The authors declare no conflicts of interest relating to the production of this manuscript.
